# Vivienne Cohen, MRCS, LRCP, FRCPsych

**DOI:** 10.1192/bjb.2021.83

**Published:** 2022-02

**Authors:** Kate Lockwood Jefford

Formerly Senior Lecturer and Consultant Medical Psychotherapist, St Bartholomew's Hospital and Medical College, London, UK

**Figure:**
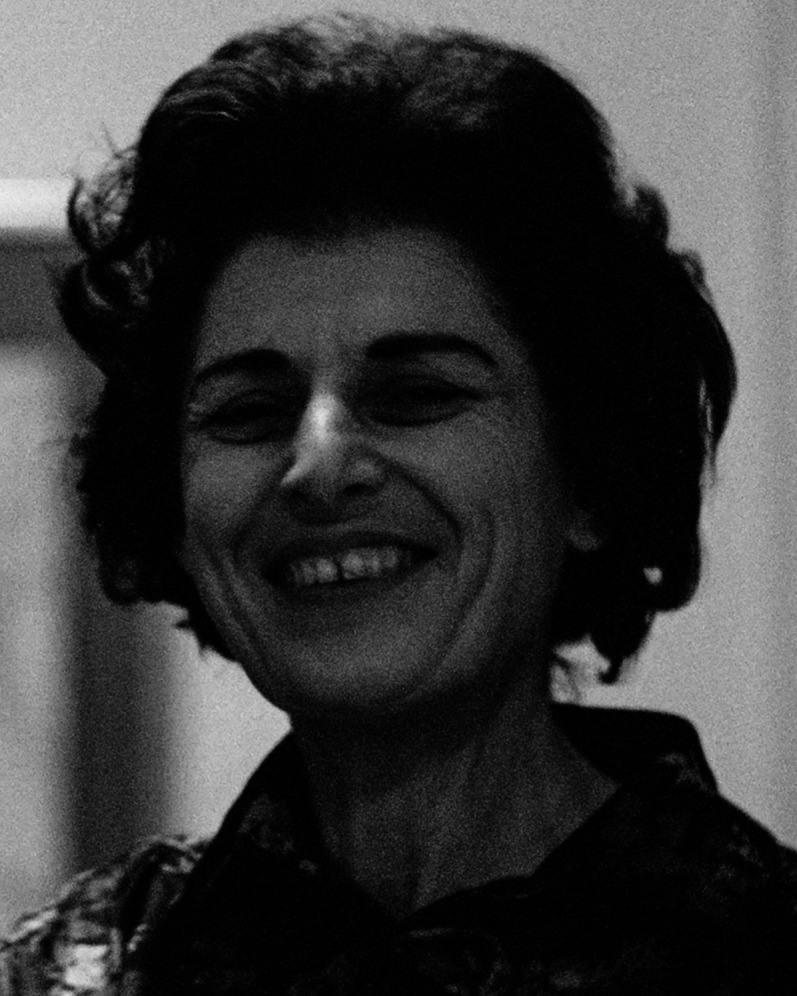

Vivienne Cohen, who has died at the age of 94, was a visionary, award-winning psychiatrist at the forefront of the developing fields of National Health Service (NHS) psychotherapy and group analytic treatments. A talented protégé of the pioneering psychoanalyst and military psychiatrist S.H. Foulkes, and the first female doctor to train in group therapy, she was a founder member of the Institute of Group Analysis (IGA) and developed one of the earliest out-patient psychotherapy departments, establishing creative links between the NHS and IGA that remain today. Her passion for training doctors and allied professionals earned her an international reputation as a teacher and supervisor.

Born Vivienne Wolfson on 1 November 1926 in London, she attended the first Jewish primary school in north-west London – co-founded by her father in 1932 – but, as war forced the family to move between London and grandparents in Hove and Ayrshire, her secondary education was fragmented across eight different schools. Vivienne studied determinedly to catch up, alongside looking after her younger sister and baby brother, early evidence of her indefatigable commitment to education and caring for others.

Her parents – and the entrenched patriarchy of the time – expected her to leave school at 14 and train as a secretary, but Vivienne had other ideas: she wanted to be a doctor. She recruited her strong-minded paternal grandmother, who argued that Vivienne was ‘too clever’ to leave school. Her parents listened.

In 1945, Vivienne won a state scholarship to University College – one of three London medical schools that accepted women – entering a pre-clinical year where one in five students were female, and completed clinical training at West London Hospital Medical School, founded to prioritise access to women. A fellow student described her as ‘principled, steady with purpose, full of ideals, brilliant and insightful, but always with a spirit of fun and humour’. In 1950 she won the Rudolf Konstan prize for medicine and she qualified in 1951.

Entering the Maudsley Hospital in 1957 as a trainee, Vivienne met Foulkes, renowned for developing innovative group treatments at the Military Neurosis Centre at Northfield. ‘Michael’, as he was known to family and close friends, became her lifelong supervisor, mentor and friend. In 1962, after working for Professor Linford Rees – a ‘marvellous’ experience where she taught and supervised psychiatric social workers – she was appointed chief assistant psychotherapist to Foulkes in his service at St Bartholomew's Hospital. After Foulkes retired in 1963 she led the service single-handedly at consultant level, expanding and improving provision to growing numbers of referrals by providing treatment both directly and from medical students and trainee psychiatrists and non-medical psychotherapists under her supervision.

In 1978 Vivienne was formally appointed consultant medical psychotherapist and senior lecturer – a rare academic post in psychotherapy – at St Bartholomew's Hospital and Medical College, and continued to build and organise the service to become one of the most reputable in the UK. In 1983 she won a national award – the first prize of this kind for psychotherapy – for her ‘imaginative’ audio-visual study of group therapy technique and practice for training purposes. In 1985 she received a Levenhulme Teaching Fellowship to support her programme of supervised clinical placements for non-medical IGA trainees. These ‘internships’ were highly competitive, as they provided a wealth of clinical experience and expert supervision, as well as enhancing the service's group treatment capacity alongside individual, couple and family therapies. In 1987 this model won her a Barnett Prize for efficient use of NHS resources.

Vivienne was a senior member of staff at the IGA and served on many local, national and international committees with an emphasis on service provision and training. She was invited frequently to teach abroad, including Denmark, Italy and Australia, and ran an intensive group therapy course in Israel. She published extensively on groups in major journals^1^ and textbooks^2,3^, covering many aspects, including supervision, training, NHS provision, cultural factors, large groups and organisational dynamics. Her vision and commercial and organisational skills played a key part in securing the building in Daleham Gardens, NW3 that is still home to the internationally renowned IGA today.

In 1955 Vivienne married Sam Cohen, a liaison psychiatrist and former professor of psychiatry at the Royal London Hospital. It was a supportive relationship with mutual regard for their respective specialties: Sam's ward milieu drew on therapeutic community principles and Vivienne published on psychological aspects of medical conditions. Sam died in London in 2004.

Vivienne eschewed private practice, preferring to work in the NHS providing what people needed, not what they could afford. Following retirement she set up a charitable foundation to fund a group psychotherapist post in the City & Hackney Psychotherapy service. When the service relocated to premises near Homerton Hospital, staff wanted a new, distinct name and, in August 2014, Vivienne Cohen House was opened at an event she attended with family, describing herself as ‘bowled over’. The chair of East London NHS Foundation Trust thanked Vivienne for her contribution to psychotherapy for residents of the City of London and Hackney over half a century, nurturing the service from its origins, sharing her skills and knowledge with patients, colleagues and trainees. Many of her trainees working today will remember their first experiences of psychotherapy supervision for her astute recall of clinical detail and her tactful creation of both personal and clinical insights in the warm ambience of her office.

Charming, vivacious and sharp, Vivienne Cohen was an independent thinker and courageous doer, a dedicated clinician and inspiring teacher. Always direct and honest, she saw herself not as a pioneer or ‘anything special’,^4^ but as someone who did what needed to be done.

Loved and admired by her children Michael and Elisheva, five grandchildren and many great-grandchildren, Vivienne was excited by the next generation, encouraging them to pursue their dreams and celebrating their achievements.

Since retirement, she divided her time between Israel and London and enjoyed opera, museums and ballet.

In January 2021, Dr Vivienne Cohen passed away at home in Israel with her family around her.
