# Obituary: Vivienne Cohen, MRCS, LRCP, FRCPsych – ERRATUM

**DOI:** 10.1192/bjb.2021.87

**Published:** 2022-02

**Authors:** Kate Lockwood Jefford

**Keywords:** Climate anxiety, climate change, psychoanalysis, post-traumatic stress disorder, ecological trauma, erratum

The Publisher would like to correct the following errors in this published Obituary:

1. In the sixth paragraph: *Levenhulme Teaching Fellowship* should instead be *Leverhulme Teaching Fellowship*.
2. In the eighth paragraph: *Sam Cohen, a liaison psychiatrist and former professor of psychiatry at the Royal London Hospital* should instead be *Sam Cohen, a liaison psychiatrist who became professor of psychiatry at the Royal London Hospital*.
3. In the eleventh paragraph: *five grandchildren and many great-grandchildren* should instead be *twelve grandchildren and twenty-nine great-grandchildren*.

The Publisher apologises for these errors.
